# Optical Model and Optimization for Coherent-Incoherent Hybrid Organic Solar Cells with Nanostructures

**DOI:** 10.3390/nano11123187

**Published:** 2021-11-24

**Authors:** Xuenan Zhao, Honggang Gu, Linya Chen, Shiyuan Liu

**Affiliations:** 1 State Key Laboratory of Digital Manufacturing Equipment and Technology, Huazhong University of Science and Technology, Wuhan 430074, China; xuenanzhao@hust.edu.cn; 2 School of Optical and Electronic Information, Huazhong University of Science and Technology, Wuhan 430074, China; linyachen@hust.edu.cn

**Keywords:** organic solar cells, optical simulation, nanostructures, incoherent substrate

## Abstract

Embedding nanostructures in organic solar cells (OSCs) is a well-known method to improve the absorption efficiency of the device by introducing the plasma resonance and scattering effects without increasing the active layer thickness. The introduction of nanostructures imposes greater demands on the optical analysis method for OSCs. In this paper, the generalized rigorous coupled-wave analysis (GRCWA) is presented to analyze and optimize the performance of coherent-incoherent hybrid organic solar cells (OSCs) with nanostructures. Considering the multiple reflections of light scattered within the glass substrate by the device, the correction vector **g** is derived, then the modified expressions for the field and absorption distribution in OSCs are provided. The proposed method is validated by comparing the simulated results of various structures with results obtained by the generalized transfer matrix method (GTMM) and the “equispaced thickness method” (ETM). The results demonstrate that the proposed method can reduce the number of simulations by at least half compared to the ETM while maintaining accuracy. With the proposed method, we discussed the device performance depending on the geometrical parameters of nanostructures, and the optimization and analysis are accomplished for single and tandem OSCs. After optimization based on the proposed method, the performance of OSCs are significantly improved, which further demonstrates the practicality of the method.

## 1. Introduction

In recent years, organic solar cells (OSCs) have gained great attention and been considered as one of the promising alternatives to produce renewable energy due to some distinct advantages such as lightweight, low cost, solution-processable, large-area manufacturing, and good mechanical flexibility [1,2,3,4]. However, the power conversion efficiency (PCE) of OSCs is still much lower than silicon solar cells, limited by the narrow absorption range and low charge mobility of organic materials [5,6]. Currently, a variety of light trapping strategies have been explored to enhance the absorption of active layers in OSCs [6,7,8,9]. Among them, embedding metallic nanostructures is one of the schemes for absorption enhancement by plasma resonance and scattering effects without increasing the active layer thickness [10,11,12,13]. Different from the silicon solar cells, OSCs usually comprise multiple functional layers on the nano-to-submicron scale and a thick substrate on the millimeter scale, which is a typical cross-scale and coherent-incoherent hybrid optical system. The performances of OSCs are highly affected by the geometrical parameters (film thickness, dimensions of nanostructures) of the device. Therefore, accurate modeling and careful design for OSCs are essential to obtain the device structure with high PCE, and the incoherency of light in the thick substrate should also be taken into account [14,15]. Additionally, systematic and abundant research is demanded for OSCs with nanostructures, especially for tandem OSCs, which is more complicated since the current match between sub-cells needs to be considered [16,17].

The optical analysis method is a powerful and useful tool to analyze and predict the device performances, as well as provide guidance on the design and optimization of OSCs [17,18,19,20,21]. Since the thickness of the substrate is much larger than the coherence length of sunlight, the optical interference effect disappears [22]. Coupled with the introduction of nanostructures, the simulation of this kind of device imposes greater demands on the optical modeling theory. There have been some methods proposed for planar OSCs or those with nanostructures. For planar OSCs, several variants based on the transfer matric method (TMM) have been proposed to consider the effect of the incoherent substrate. In the generalized transfer matrix method (GTMM) [23,24], the light propagation in the incoherent substrate is separately described by the modified intensity matrix similar to the TMM. There are also a series of averaging methods, such as random phase method (RPM) [25] and equidistant phase method (EPM) [26], introducing the incoherence by averaging the TMM calculation results with random/equidistant phases shifts added to the incoherent layer. Additionally, Marko et al. combined TMM with ray tracing to propose a simulator called the Combined Ray Optics/Wave Optics Model (CROWN) and suggested that the rigorous coupled-wave analysis (RCWA) could also be combined with ray tracing [27]. For non-planar OSCs, some numerical simulation methods, such as RCWA [28], the finite element method (FEM) [29], and the finite-difference time-domain (FDTD) [16,30], can be used to simulate the coherent region in OSCs with nanostructures, and an additional procedure to handle the incoherency of light in the thick substrate is needed. There have been various methods to deal with the coherent-incoherent hybrid non-planar condition, including the spectral averaging method (SAM) [31], the first-principle calculation [32], and one-pass coherent calculation [33], requiring additional mathematical and computational processes which increased the complexity and time for calculation. Additionally, there are also several averaging methods, such as the phase elimination method (PEM) [34], and equispaced thickness method (ETM) [29], considering the incoherence by averaging the FEM or FDTD simulation results with multiple additional thickness thicknesses added to the incoherent layer. In the PEM, only two coherent calculation results with adjusted thickness are averaged to eliminate the interference in the incoherent substrate. However, the PEM requires a very tiny discretization step to reduce the phase error, which will increase the computation time. In the ETM, nearly ten coherent results are required to be averaged for acceptable accuracy. In addition, high-order multiple reflection terms in the incoherent substrate are ignored in the PEM and ETM calculation, which would cause additional calculation errors.

In this paper, we proposed a generalized rigorous coupled-wave analysis (GRCWA) method to simulate the coherent-incoherent hybrid OSCs with nanostructures. In the GRCWA method, the incoherency of light in the substrate is considered via the introduced **g** vector, which can modify the RCWA calculation results for the coherent region. Taking the approach for one-dimension nanostructures as an example, the thorough description of the GRCWA, including the mathematical procedures and the physical background will be represented detailed in Section 2. Then in Section 3, the GRCWA will be applied to various OSCs and compared with other simulation techniques to verify the accuracy and superiority of the proposed method. The reflectivity spectra simulated by the proposed method are in good agreement with other methods. In addition, the analysis and optimization based on the GRCWA are carried on the single and tandem OSCs with nanostructures, respectively, which shows the ability of the method to design OSCs with high efficiency.

## 2. Theory and Methods

**Figure 1:**
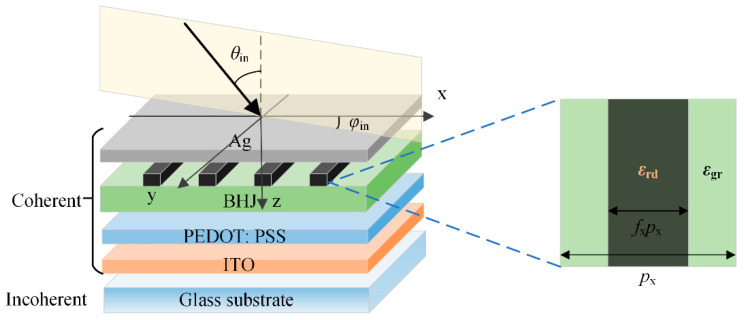
Schematic diagram of the stratified structure of the OSC with one-dimensional metal nanogratings. (The structure of OSC with two-dimensional nanogratings are shown in Figure S1.)

The proposed GRCWA is a generic form based on RCWA, incorporating the BSDF theory and introducing a **g** vector, so as to include the incoherent thick layer and nanostructured layer reflection and transmission. The GRCWA can be employed in the coherent-incoherent hybrid system, both for planar and non-planar devices. In this section, the GRCWA formalisms are given for an OSC with a one-dimensional nanostructure as an example (the detailed process for OSCs with two-dimensional nanostructures is shown in the Supplementary Materials). Figure 1 shows a typical structure of a single-junction OSC device with nanostructured layers, consisting of an incoherent glass substrate and coherent multilayers between the semi-infinite transparent ambient on the top and bottom. Each layer *j* has a thickness *d_j_* and a dielectric function *ε_j_*. The dielectric function *ε_j_* can be derived from the complex refractive index *N_j_* = *n_j_* − j*κ_j_* according to the following equations

(1) $$\varepsilon_{j}=N_{j}{}^{2}=n_{j}{}^{2}-2n_{j}\kappa_{j}j-\kappa_{j}{}^{2},$$

where, *n* and *κ* are refractive index and extinction coefficient, respectively, and j is the imaginary unit. We assume that the unpolarized light propagates from the glass side at the incident angle *θ*_in_ and azimuthal angle *φ*_in_. We consider the thick glass substrate as an incoherent layer because its thickness (~700 μm) is much larger than the coherence length of sunlight (~0.6 μm) [22], while the remaining layers with thin thicknesses are regarded as coherent layers. Taking the nanostructured OSCs in Figure 1 as examples, the one-dimensional metal nanogratings are introduced at the interface between the active layer and the cathode. The periods and duty cycles of nanogratings are *p* and *f*, respectively. The dielectric functions of the materials in the grating area and the filling area are *ε*_rd_ and *ε*_gr_, respectively. The dielectric function can also be thought of as several one-dimensional periodic functions; hence the one-dimensional Fourier expansion can be performed on the dielectric function

(2) $$\varepsilon \left(x\right)=\sum_{v=-\infty }^{\infty }\varepsilon_{v}\exp\left(j\frac{2\pi v}{p}x\right),$$

where the Fourier coefficient is

(3) $$\varepsilon_{v}=\frac{1}{p}\int_{-p/2}^{p/2}\varepsilon \left(x\right)\exp\left(-j\frac{2\pi v}{p}x\right)dx.$$

The integers *v* indicates the expansion orders.

### 2.1. Optical Model Based on the RCWA

**Figure 2:**
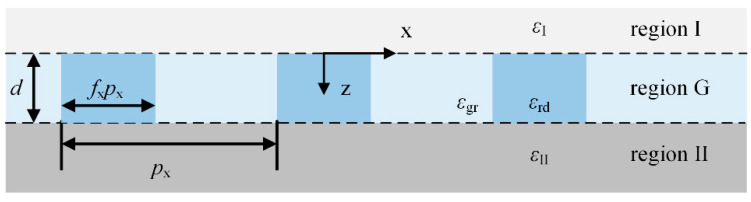
Schematic diagram of the periodic grating structure in the optical model. *ε*_I_ and *ε*_II_ are the dielectric functions of the materials in the incident and transmittance region respectively. *ε*_rd_ and *ε*_gr_ are the dielectric functions of the materials in the grating area and the filling area respectively.

For the coherent region in OSC, the behavior of light propagation can be modeled based on the RCWA. As shown in Figure 2, for the periodic nanostructured layer, there exist three regions, namely the incident region I, the transmittance region II, and the grating region G. According to the Floquet condition [35], the electric and magnetic fields in region G can be described as

(4) $$E(x,y,z)=\sum_{m=-\infty }^{\infty }\left[S_{x}^{m}(z)\;\hat{x}+S_{y}^{m}(z)\;\hat{y}+S_{z}^{m}(z)\;\hat{z}\right]\exp\left[-j\left(k_{xm}x+k_{y}y\right)\right],$$

(5) $$H(x,y,z)=\sum_{m=-\infty }^{\infty }\left[U_{x}^{m}(z)\;\hat{x}+U_{y}^{m}(z)\;\hat{y}+U_{z}^{m}(z)\;\hat{z}\right]\exp\left[-j\left(k_{xm}x+k_{y}y\right)\right],$$

where *S* and *U* are the components of the electric and magnetic fields respectively, *m* is the diffraction order at the x-direction, and *k*_x*m*_ = *k*_0_[*n*sin*θ*_in_cos*φ*_in_ − *m*(*λ*/*p*)] and *k*_y_ = *k*_0_*n*_I_-sin*θ*_in_sin*φ*_in_) are the x- and y-components of the wave vector, respectively.

Substituting Equations (4) and (5) into Maxwell’s equations and eliminating the z-components of the electric and magnetic fields, we can obtain a set of differential equations for *S*_x_, *S*_y_, and *U*_x_, *U*_y_:

(6) $$\frac{\partial }{\partial z^{\prime }}\left[\begin{array}{c} S_{y}\\ S_{x}\\ U_{y}\\ U_{x} \end{array}\right]=\left[\begin{array}{cc} 0 & P\\ Q & 0 \end{array}\right]\left[\begin{array}{c} S_{y}\\ S_{x}\\ U_{y}\\ U_{x} \end{array}\right].$$

Herein, **S**_x_, **S**_y_, **U**_x_, and **U**_y_ are *M* × 1 vectors composed of $S_{x}^{m}$, $S_{y}^{m}$, $U_{x}^{m}$ and $U_{y}^{m}$, and *M* = 2*T*_x_ + 1 is the number of terms in the Fourier expansion of the electromagnetic field, *T*_x_ is the truncation orders for Fourier expansion. After eliminating **U**_x_, **U**_y_ or **S_x_**, **S**_y_, the set of differential equations become a set of partial differential equations, which can be written in matrix form as

(7) $$\frac{\partial^{2}}{\partial {z^{\prime }}^{2}}\left[\begin{array}{c} S_{y}\\ S_{x} \end{array}\right]=PQ\left[\begin{array}{c} S_{y}\\ S_{x} \end{array}\right],$$

(8) $$\frac{\partial^{2}}{\partial {z^{\prime }}^{2}}\left[\begin{array}{c} U_{y}\\ U_{x} \end{array}\right]=QP\left[\begin{array}{c} U_{y}\\ U_{x} \end{array}\right].$$

Here, **PQ** and **QP** are 2*M* × 2*M* matrices. Solving the partial differential equations, we get

(9) $$\left[\begin{array}{c} S_{y}\\ S_{x}\\ U_{y}\\ U_{x} \end{array}\right]=\left[\begin{array}{cc} W & W\\ -V & V \end{array}\right]\left[\begin{array}{cc} e^{-k_{0}qz} & 0\\ 0 & e^{k_{0}q\left(z-d\right)} \end{array}\right]\left[\begin{array}{c} C^{+}\\ C^{-} \end{array}\right],$$

where **W** and **V** are 2*M* × 2*M* matrices composed of eigenvectors of **PQ** and **QP**, **q** is a 2*M* × 2*M* diagonal matrix consisting of the square roots of the eigenvalues of **PQ**, **C**^+^, and **C**^−^ are the field coefficients vectors, which can be solved with the boundary conditions that the tangential field components are continuous.

At the upper and lower interfaces of the *γ*-th layer, the boundary conditions exist

(10) $$\left[\begin{array}{cc} W_{\gamma -1}X_{\gamma -1} & W_{\gamma -1}\\ -V_{\gamma -1}X_{\gamma -1} & V_{\gamma -1} \end{array}\right]\left[\begin{array}{c} C_{\gamma -1}^{+}\\ C_{\gamma -1}^{-} \end{array}\right]=\left[\begin{array}{cc} W_{\gamma } & W_{\gamma }X_{\gamma }\\ -V_{\gamma } & V_{\gamma }X_{\gamma } \end{array}\right]\left[\begin{array}{c} C_{\gamma }^{+}\\ C_{\gamma }^{-} \end{array}\right],$$

(11) $$\left[\begin{array}{cc} W_{\gamma }X_{\gamma } & W_{\gamma }\\ -V_{\gamma }X_{\gamma } & V_{\gamma } \end{array}\right]\left[\begin{array}{c} C_{\gamma }^{+}\\ C_{\gamma }^{-} \end{array}\right]=\left[\begin{array}{cc} W_{\gamma +1} & W_{\gamma +1}X_{\gamma +1}\\ -V_{\gamma +1} & V_{\gamma +1}X_{\gamma +1} \end{array}\right]\left[\begin{array}{c} C_{\gamma +1}^{+}\\ C_{\gamma +1}^{-} \end{array}\right].$$

Here, **X***_γ_* is the diagonal matrix, composed of exp(−*k*_0_*q_γ_*_, *i*_*d_γ_*), *q_γ_*_,*i*_ is the positive square root of the eigenvalue of **P***_γ_***Q***_γ_*. For the incident and transmitted interfaces of the coherent multilayers, the boundary conditions are

(12) $$\left[\begin{array}{c} S_{0}{}_{x}\\ S_{0}{}_{y}\\ U_{0}{}_{x}\\ U_{0}{}_{y} \end{array}\right]+\left[\begin{array}{c} I\\ \zeta \end{array}\right]\left[\begin{array}{c} r_{x}\\ r_{y} \end{array}\right]=\left[\begin{array}{cc} W_{1} & W_{1}X_{1}\\ -V_{1} & V_{1}X_{1} \end{array}\right]\left[\begin{array}{c} C_{1}^{+}\\ C_{1}^{-} \end{array}\right],$$

(13) $$\left[\begin{array}{cc} W_{\mathrm{end}}X_{\mathrm{end}} & W_{\mathrm{end}}\\ -V_{\mathrm{end}}X_{\mathrm{end}} & V_{\mathrm{end}} \end{array}\right]\left[\begin{array}{c} C_{\mathrm{end}}^{+}\\ C_{\mathrm{end}}^{-} \end{array}\right]=\left[\begin{array}{c} I\\ \varsigma \end{array}\right]\left[\begin{array}{c} t_{x}\\ t_{y} \end{array}\right],$$

**S**_0_ and **U**_0_ are the x- and y-components of the incident electric and magnetic fields respectively. The matrices **ζ** and **ς** depend on the kind of diffraction and can be calculated from the wave vector components. **r**_x_, **r**_y_, **t**_x_, and **t**_y_ are the reflected amplitude vectors and transmitted amplitude vectors at x and y directions, respectively.

Solving the system of equations formed by the above boundary conditions, the field coefficient **C**^+^, **C**^−^ and the reflected, transmitted field amplitudes **r**, **t** can be obtained. Then, the amplitudes of the electromagnetic field components inside the device can be obtained.

### 2.2. Optical Model for the Hybrid Coherent-Incoherent OSCs Based on BSDF

**Figure 3:**
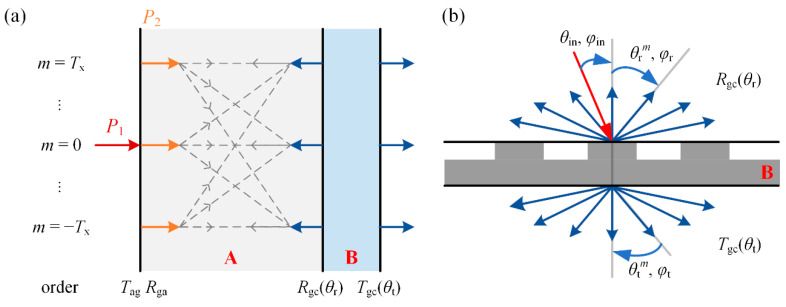
(**a**) Optical model of OSCs with nanostructured layer based on BSDF. Regions A and B are the incoherent glass substrate and the coherent multilayers containing the nanostructured layers, respectively. The gray dashed lines indicate the contribution of the reflected light at glass/air and A/B interface to each other. The reflected light of the glass/air interface at a particular order can contribute to the multi-orders reflected light at the A/B interface. While the reflected light of the A/B interface at a particular order can only contribute to the reflected light of the glass/air interface at the same order. (**b**) Schematic diagram of the BSDF model for region B with nanostructured layers in Figure 3a. (The details for OSC with two-dimensional nanogratings are shown in Figure S2.)

As shown in Figure 3, the device is divided into two parts due to the thick glass substrate. Regions A and B indicate the incoherent glass substrate and coherent region, respectively. Due to the scattering effect of the nanostructures embedded in region B, the reflected light inside region A consists of light in multiple directions, and the light undergoes infinite reflections between the upper and lower interfaces of the substrate. As a result, the incident light for the coherent region B propagates in multiple directions. Because of the disappearance of the optical coherence in the substrate, we use the bidirectional scattering distribution functions (BSDF) to describe the intensity change as the light propagates. At the wavelength *λ*, the sunlight with intensity *P*_1_ transmits from air to the device. *T*_ag_ and *R*_ag_ is the transmittance and reflectance of light from the air to the glass substrate. *T*_ga_ and *R*_ga_ are the transmittance and reflectance of light from the glass to the air. *R*_gc_(*θ*_r_) and *T*_gc_(*θ*_t_) are the BSDF from the glass to the coherent region B, which can be calculated from the reflectance and transmission coefficients in Section 2.1. The details of the BSDF are shown in Figure 3b. Each set of wave vectors with wavelength *λ* has *M* orders, in other words, there is light in *M* directions contemplated. Representing the propagation of light in multiple directions in matrix or vector form, *P*_1_ can be written as **P**_1_, a column vector of *M* × 1. The incident energy of all directions is zero except for the light at *θ*_in_ where the incident energy is 1. Correspondingly, *R*_gc_(*θ*_r_) and *T*_gc_(*θ*_t_) can be described as two *M* × *M* square matrices **R**_gc_ and **T**_gc_, the column vectors of **R**_gc_ and **T**_gc_ are the reflectance and transmission vectors of light incident at a certain direction. *R*_ga_ and *T*_ga_ can be written as two *M* × *M* diagonal matrices **R**_ga_ and **T**_ga_, the diagonal elements are reflectance or transmittance from the glass to air at all diffractive directions. The incident angle and azimuthal angle for each diffraction order are

(14) $$\theta_{r}^{m}=\arcsin\left[\frac{1}{n_{g}}\left(n_{a}\sin\theta_{\mathrm{in}}\cos\varphi_{\mathrm{in}}-m\frac{2\pi }{p}\right)\right],$$

(15) $$\theta_{t}^{m}=\arcsin\left[\frac{1}{n_{a}}\left(n_{a}\sin\theta_{\mathrm{in}}\cos\varphi_{\mathrm{in}}-m\frac{2\pi }{p}\right)\right],$$

(16) $$\varphi_{r}=\arcsin\left[\frac{1}{n_{g}}\left(n_{a}\sin\theta_{\mathrm{in}}\sin\varphi_{\mathrm{in}}\right)\right],$$

(17) $$\varphi_{t}=\arcsin\left[\frac{1}{n_{a}}\left(n_{a}\sin\theta_{\mathrm{in}}\sin\varphi_{\mathrm{in}}\right)\right],$$

where *n*_a_ and *n*_g_ are refractive indices of the air and glass substrate, respectively. As each of the above angles has a maximum value of π/2, the diffraction order *m* is somewhat limited. In other words, only diffracted light under *M*′ order can be reflected in the glass substrate. As a result, the number of reflected light directions considered in the calculation *M*′ can be smaller than the total number of diffraction order *M* in the RCWA calculation, which is usually set as a higher value to ensure convergence. Hence the above vectors and matrices can be reduced in dimension from *M* to *M*′.

Since there is a scattering effect in region B, the light in region A will propagate in multiple directions. The light will be multi-reflected at the glass/air interface and A/B interface, and light at different orders will contribute to each other as the gray dashed line shows in Figure 3a. The incident intensity **P**_2_ from region A to region B can be described as a collection of light intensities in multiple directions as

(18) $$\begin{array}{cc} P_{2} & =T_{\mathrm{ag}}P_{1}+T_{\mathrm{ag}}R_{\mathrm{gc}}R_{\mathrm{ga}}P_{1}+T_{\mathrm{ag}}{\left(R_{\mathrm{gc}}R_{\mathrm{ga}}\right)}^{2}P_{1}+\cdots \\ & =T_{\mathrm{ag}}{\left[I-R_{\mathrm{gc}}R_{\mathrm{ga}}\right]}^{-1}P_{1}, \end{array}$$

where **I** is a *M*′ × *M*′ unit matrix and **P**_2_ is a *M*′ × 1 vector.

Similarly, the reflectivity **R** of the whole device is

(19) $$R=R_{\mathrm{ag}}P_{1}+\frac{T_{\mathrm{ag}}T_{\mathrm{ga}}R_{\mathrm{gc}}}{I-R_{\mathrm{gc}}R_{\mathrm{ga}}}P_{1}$$

### 2.3. Field and Power Dissipation and Performance Parameters of OSCs

For lossless mediums such as the air and the glass substrate, the intensity *P* is given by

(20) $$P=\frac{1}{2}\varepsilon_{0}cn\cos\theta {\left|E\right|}^{2},$$

where *E* is the electric field amplitude in the medium, c and ε_0_ are the light speed and the permittivity in free space respectively, *n* is the refractive index of the medium, and *θ* indicates the direction of light propagation. According to Equations (18) and (20), the incident electric field amplitudes from the glass substrate at the *i*-th direction *E*_g*i*_ and the initial incident electric field amplitude from the air *E*_a_ are related by a glass factor *g_i_* as follows

(21) $${\left|E_{gi}\right|}^{2}={\left|g_{i}\right|}^{2}\cdot {\left|E_{a}\right|}^{2}.$$

The glass vector **g** is a *M*′ × 1 vector composed of *g_i_*, and can be obtained by

(22) $$g\circ g=\frac{n_{a}\cos\theta_{\mathrm{in}}T_{\mathrm{ag}}\cdot I\cdot P_{1}}{n_{g}C_{g}\left(I-R_{\mathrm{ga}}R_{\mathrm{gc}}\right)},$$

where the operator symbol “⸰” means to solve the Hadamard product. **C**_g_ is a *M*′ × *M*′ diagonal matrix, with cos(*θ*_g_) at different directions as the diagonal elements, *θ*_g_ is the incident angle from the glass to the coherent multilayers.

As non-zero order incident light intensity is not necessarily zero, the contribution of non-zero order incident light should be considered when calculating the electromagnetic field in the device. Consequently, the amplitudes of the electric and magnetic field components at each order in Equation (4) should be written as

(23) $$S_{x}^{m}(z)=\sum_{i=1}^{M^{\prime }}g_{i}S_{xi}^{m}(z),$$

(24) $$U_{x}^{m}(z)=\sum_{i=1}^{M^{\prime }}g_{i}U_{xi}^{m}(z).$$

Here, $S_{xi}^{m}$ and $U_{xi}^{m}$ are the x-components of the electric and magnetic field amplitudes for each diffraction order contributed by the *i*-th direction incident light, and can be calculated assuming that the incident light intensity at the *i*-th direction is 1. The amplitude of the electric and magnetic field components in other directions is calculated in the same way.

According to the calculated electromagnetic field distribution, the optical power dissipation at any position is

(25) Q(x,y,z)=12cε0k0Im(ε(x,y,z))|E(x,y,z)|2,

where, *k*_0_ is the wave vector in the air, *k*_0_ = 2π/*λ*. The photogenerated carrier rate is

(26) $$G\left(x,y,z\right)=\frac{2{\pi \varepsilon }_{0}n\left(x,y,z\right)\kappa \left(x,y,z\right)P_{\mathrm{in}}}{h}{\left|E\left(x,y,z\right)\right|}^{2}.$$

Finally, the absorptance of the absorber can be written as

(27) $$A=\frac{1}{P_{0}}{∭}_{V}Q\left(x,y,z\right)dxdydz,$$

where *P*_0_ is the incident optical power of sunlight and *V* is the volume of the absorber.

Equations (23)–(27) provide the expressions for the optical power flow and absorption characteristics in OSCs containing incoherent layers and nanostructured layers.

Based on the above model, the short-circuit current density *J*_SC_ generated by the device can be evaluated as

(28) $$J_{\mathrm{SC}}=\eta_{\mathrm{IQE}}\cdot \int \frac{q\lambda }{\mathrm{hc}}P_{\mathrm{in}}\left(\lambda \right)A_{\mathrm{ac}}\left(\lambda \right)d\lambda ,$$

where *η*_IQE_ is the internal quantum efficiency of the device, usually assumed to be 1, q is the unit charge, and *A*_ac_ is the absorptance of the active layers.

While for the tandem OSCs, sub-cells have the same current density according to Kirchhoff’s law since they are connected in series. The *J*_SC_ of the whole tandem device is smaller than those in the sub-cells,

(29) $$J_{\mathrm{SC}}=\min\left(J_{\mathrm{SC}\text{-}f},J_{\mathrm{SC}\text{-}r}\right),$$

where *J*_SC-f_ and *J*_SC-r_ are the short-circuit current density of the front and rear sub-cell, calculated by Equation (28) from the absorptances of the front and rear active layers.

## 3. Results and Discussion

### 3.1. Planar Single-Junction OSCs

**Figure 4:**
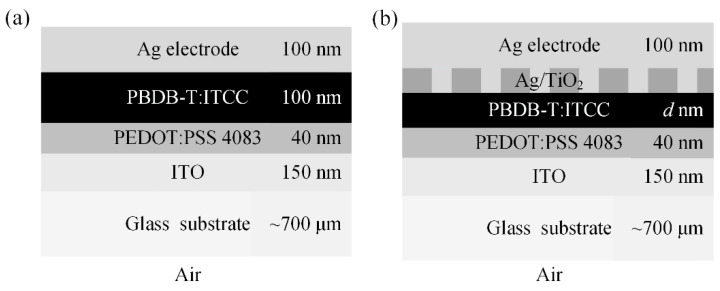
Schematic diagram of the device structures: (**a**) the planar single-junction OSC; (**b**) the single-junction OSC with a nanostructured layer.

**Figure 5:**
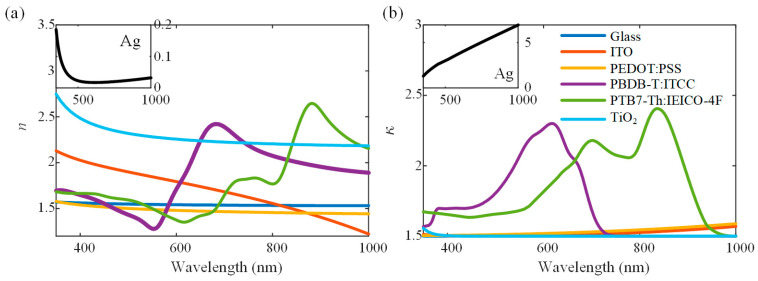
(**a**) Refraction index (*n*) and (**b**) extinction coefficient (*κ*) of materials employed in the investigated OSCs. PTB7-Th was purchased from 1-Material Inc. PBDB-T, ITCC, and IEICO-4F were purchased from Solarmer. PEDOT: PSS (Clevios P VP Al 4083) was purchased from Heraeus. Unless otherwise stated, all reagents and solvents were commonly commercially available products and were used as received. Both the PBDB-T: ITCC (*w*:*w*= 1:1) and PTB7-Th: IEICO-4F (*w*:*w*= 1:1.5) solutions were prepared in a mixed solvent chlorobenzene (CB): 1,8-diiodooctane (DIO) (*v*:*v* = 99.5: 0.5 for PBDB-T:ITCC, *v*:*v* = 99: 1 for PTB7-Th: IEICO-4F). The detailed preparation of these materials has been presented in our previous work [17].

The planar single-junction OSC structure is shown in Figure 4a, which is composed of a 700 μm thick glass substrate, a 150 nm ITO layer as the anode layer, a 40 nm PEDOT: PSS layer as the hole transport layer, a 100 nm PBDB-T: ITCC as the active layer, and a 100 nm Ag as the cathode layer. The unpolarized sunlight is incident on the device from the glass substrate side at an incident angle of *θ*_in_. The thick glass substrate is considered an incoherent layer. Figure 5 shows the optical constants (including the refractive index *n* and extinction coefficient *κ*) of materials used in the OSCs, obtained by the spectroscopic ellipsometry or previous publications [17,36]. To validate the proposed GRCWA methods, we compared simulation results of the planar single OSC based on the GRCWA model with those calculated by the analytical GTMM model.

**Figure 6:**
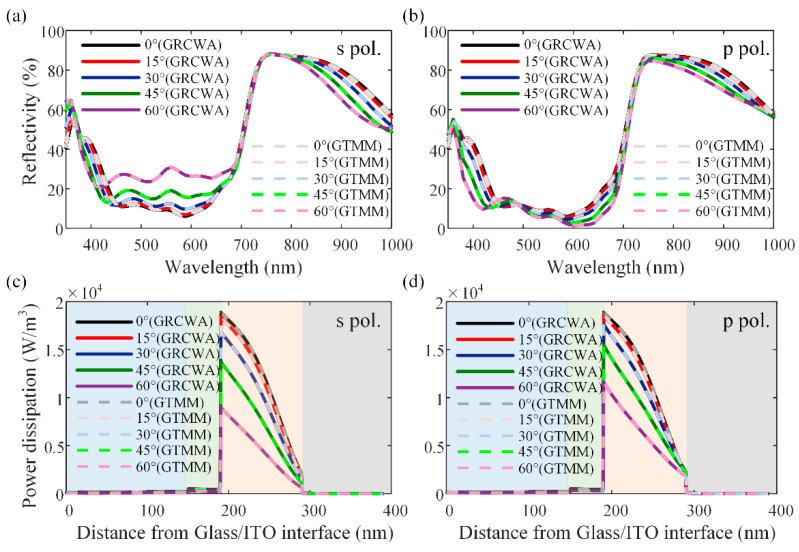
Comparison of the simulation results of the device shown in Figure 3a using the GRCWA and the GTMM methods at different incident angles and polarizations: (**a**,**b**) the reflectivity spectra for s- and p-polarized light respectively; (**c**,**d**) spatial power dissipation profiles at the wavelength of 600 nm for s- and p-polarized light respectively.

Figure 6a,b show the simulated reflectivity spectra of the planar single OSC with the varying incident angle for the s- and p-polarized light. Figure 6c,d show the simulated spatial profiles of the power dissipation (*Q*) in the device with the varying incident angle and polarization at the wavelength of 600 nm. Different color blocks from left to right indicate the ITO, PEDOT: PSS, PBDB-T: ITCC, and Ag layers, respectively. At normal incidence, the power dissipation and reflectivity are identical for the s- and p-polarizations. As the incident angle increases, the optical power dissipations in the active layer decrease for both polarizations, but the device shows comparatively higher absorption for the p-polarized light than the s-polarized light. The above results show that the spatial absorption profile and the reflectivity calculated by the proposed GRCWA are well-matched with the results obtained by the GTMM at all the incident angles and polarizations of incident light. Meanwhile, the proposed GRCWA is also able to deal with the case for non-planar OSCs, which is not available with GTMM. Results on the applications of GRCWA on non-planar OSCs will be given and discussed below.

### 3.2. Single-Junction OSCs with Nanogratings

**Figure 7:**
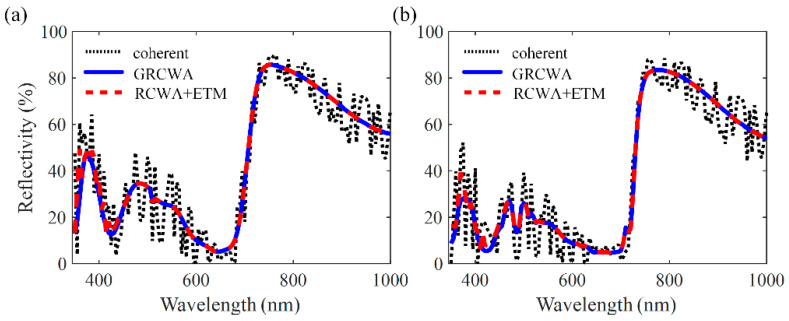
Calculated reflectivity spectra at (**a**) s- and (**b**) p-polarization of the device shown in Figure 3b (*d* = 50 nm, *p* = 300 nm, *w* = 100 nm, *h* = 20 nm) using the coherent, the GRCWA, and the ETM methods.

Figure 4b shows the device structure of the single-junction OSC with nanogratings. To enhance the absorption of the active layer, the one-dimensional rectangular wire nanogratings with a period *p*, width *w*, and height *h* are introduced. The troughs of the nanogratings are filled with TiO_2_, which is the best electron transport material [37]. To further verify the correctness and superiority of the proposed method, the simulations based on the GRCWA and ETM are both carried on the single nanostructured OSCs at normal incidence. The thickness of the PBDB-T: ITCC active layer is set as *d* = 50 nm. The geometrical parameters of gratings are set as *p* = 300 nm, *w* = 100 nm, and *h* = 20 nm. The truncation orders of RCWA calculations are both 10. In the ETM calculation, the RCWA simulation results with eight equispaced thicknesses are averaged. Additionally, according to the wavelength and period of the nanostructure, the number of reflected light directions considered in the GRCWA calculation is three (order = −1, 0, 1). Figure 7a,b show the simulated reflectivity spectra of the single OSCs for the s- and p-polarized light. When the thick glass substrate is considered perfectly coherent, the calculation results show a lot of reflection ripples. When the two incoherent methods are applied to the simulation, the reflection ripples disappear, and the calculation results of the two methods are in good agreement. According to the deviations study of ETM in [29], the number of RCWA simulations with corresponding equispaced thicknesses should be larger than seven to ensure the accuracy of ETM. While for the proposed GRCWA, the number of RCWA simulations is decided by the wavelength range and period of nanostructures and usually does not exceed five in OSC simulations. The results show that the proposed GRCWA can be applied to the nanostructured OSCs and provide acceptable accuracy with a lower number of simulations.

**Figure 8:**
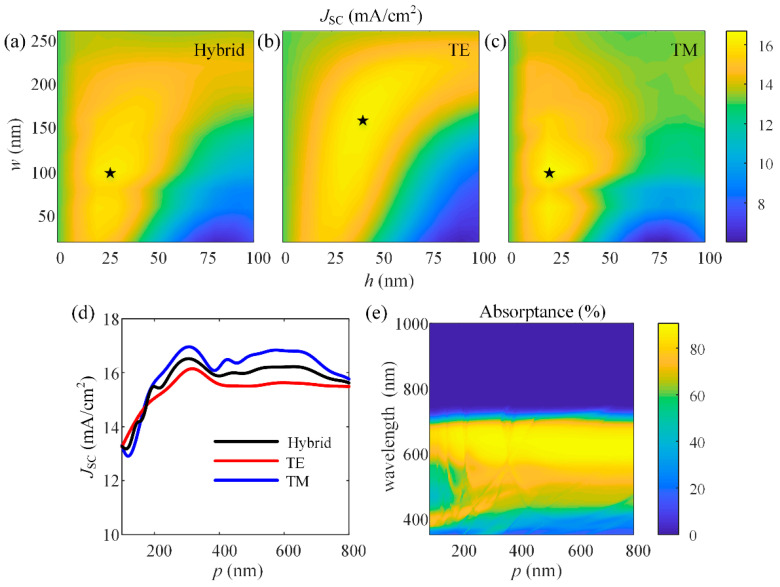
Simulated *J*_sc_ generated in the device as a function of the width *w* and height *h* of the nanogratings (*p* = 300 nm) at (**a**) hybrid, (**b**) TE, and (**c**) TM polarization (The star sysmbol in each subgraph represents the optimal structures at the corresponding polarization condition); (**d**) simulated *J*_sc_ generated in the OSC and (**e**) simulated absorption spectrum of the active material as a function of the period *p* of the nanogratings (*w* = 100 nm, *h* = 25 nm).

Furthermore, the optical analysis and optimization are performed on the single OSCs using the proposed method. The short-circuit current *J*_SC_ is simulated as functions of the geometrical parameters (*w*, *h*, and *p*) at the normal incidence and the *J*_SC_ is calculated via integrating the photons absorbed over the wavelength range from 350 nm to 1000 nm. Figure 8a–c show the dependence of the *J*_SC_ on the width *w* and height *h* of the nanogratings at different polarizations. In this simulation, the period *p* is set as 300 nm, the height *h* varies from 0 to 100 nm with the step set as 5 nm, and the width *w* varies from 0 to 260 nm with the step set as 10 nm. It can be seen that the *J*_SC_ demonstrates interference oscillations with *w* and *h* changing. From Figure 8c, we can find that the *J*_SC_ shows stronger dependence on the geometrical parameters (*w* and *h*) at the TM polarization, and it reaches a maximum of 16.65 mA/cm^2^ when *w* = 100 nm and *h* = 20 nm. While at the TE polarization, the *J*_SC_ reaches a maximum of 16.91 mA/cm^2^ when *w* = 160 nm and *h* = 40 nm as shown in Figure 8b. As a result, for the hybrid unpolarized incident light, the maximum *J*_SC_ is 16.46 mA/cm^2^ at a width of 100 nm and a height of 25 nm, as shown in Figure 8a. The dependence of the *J*_SC_ on the period *p* of the nanogratings is shown in Figure 8d, the width *w* and height *h* are set as the optimal size (*w* = 100 nm, *h* = 25 nm) in Figure 8a. It can be observed that when the period is longer than 180 nm, larger *J*_SC_ is generated at the TM polarization than at the TE polarization. For unpolarized light, the value of *J*_SC_ maintains a relatively high level for a period longer than 240 nm, corresponding to the absorption spectra shown in Figure 8e, where the active layer effectively absorbs light in the wavelength region from 410 nm to 710 nm. Additionally, the *J*_SC_ reaches a maximum 16.53 mA/cm^2^ for unpolarized light when *p* = 330 nm, which is increased 25.5% over the *J*_SC_ = 13.17 mA/cm^2^ for the device without nanogratings. The optimized results for OSCs with different active layer thicknesses are summarized in Table 1. It can be seen that the absorption enhancement of nanogratings is more effective for the device with a thinner active layer (as shown in Table 1).

**Figure 9:**
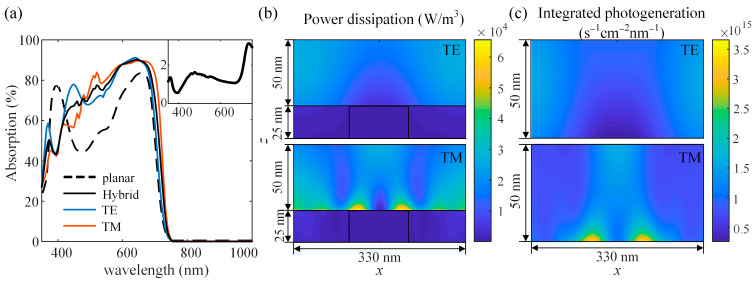
Performances of the single-junction OSCs: (**a**) absorption spectra of active layers in devices with and without nanogratings, and the inset shows the ratio of absorption by the nanostructured OSC and the planar OSC; (**b**) distribution of the power dissipation in the active layer and nanogratings at the wavelengths of 520 nm; (**c**) distribution of the integrated photogeneration rate in the active layer over the wavelength from 350 nm to 1000 nm.

Figure 9 shows the performances of the planar and non-planar single-junction OSCs. The active layer thickness is set as *d* = 50 nm and the geometrical parameters of nanogratings are set based on the optimization results above (*w* = 100 nm, *h* = 25 nm, *p* = 330 nm). The absorption spectra of the active layers in devices with and without nanogratings are shown in Figure 9a. It can be seen that the nanostructured back cathode provides obvious absorption enhancement at the wavelength from 420 nm to 750 nm, especially for the region near the bandgap. Several additional absorption peaks appear at wavelengths of 370 nm, 450 nm, 520 nm, and 640 nm. We plotted the distribution of power dissipation *Q*(*x*, *y*, *z*) at a wavelength of 520 nm as shown in Figure 9b. At the TM polarization, due to the excitation of surface plasmon polaritons (SPP), strong absorption can be observed in the vicinity of the Ag grating surface, and the power dissipation *Q* shows stronger variations along the x-axis at the TM polarization than that at the TE polarization. Although *Q* is weaker at the TE polarization than that for the case at the TM polarization, it is still enhanced due to the scattering and trapping effects introduced by the nanogratings compared to the planar OSC. Consistent with Figure 9a, the nanostructured back cathode has greater enhancement effects on the absorption at the TM polarization at the wavelength of 520 nm. In Figure 9c, the photogeneration rate *G* in the active layer is integrated over the wavelength from 350 nm to 1000 nm. Additionally, it is clear that a large number of carriers are generated in the vicinity of the Ag grating surface at the TM polarization. As a result, the *J*_SC_ generated in the device increased significantly from 13.17 to 16.53 mA/cm^2^. These results demonstrate that the introduction of nanogratings in single-junction OSCs can improve the device performance significantly, especially for a device with a thin active layer, avoiding increasing the active layer thickness, which would cause some unfavorable situations such as carriers combination and reduced carrier mobility.

### 3.3. Tandem OSCs with Nanostructures

**Figure 10:**
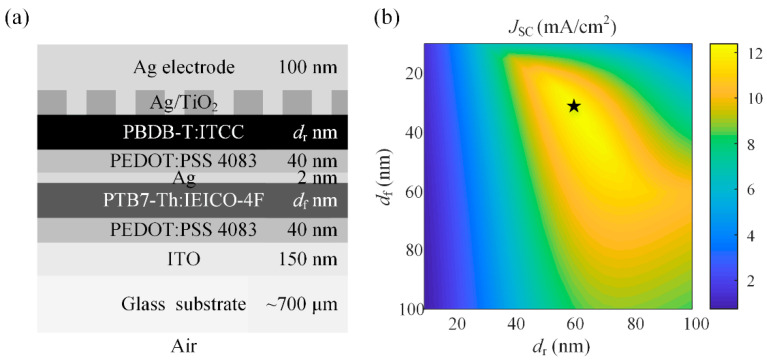
(**a**) The device structure of the tandem OSC with one-dimensional rectangular wire nanogratings; (**b**) *J*_SC_ of the planar tandem OSC as functions of thicknesses of the front and rear active layers at normal incidence. (The star sysmbol represents the optimal structure).

To investigate the effect of the nanostructures on the performances of tandem OSCs, we simulated a tandem OSC with the structure given in Figure 10a. PTB7-Th: IEICO-4F and PBDB-T: ITCC are chosen as the active materials for the front and rear sub-cells, respectively. It can be seen from Figure 5b that the front-cell active layer PTB7-Th: IEICO-4F with a bandgap of 1.25 eV can absorb light over the visible and infrared range especially from 550 nm to 1000 nm. While the rear-cell active layer PBDB-T: ITCC with a bandgap of 1.66 eV mainly absorbs light over the visible range from 350 nm to 740 nm. Usually, the performance of tandem OSC is optimized by adjusting the thicknesses of two active layers [17]. For the planar tandem OSC, we simulated the *J*_SC_ varied with the thicknesses of two active layers at normal incidence in Figure 10b. The *d*_f_ and *d*_r_ are both varied from 10 to 100 nm. It is obvious that *J*_SC_ shows the interference oscillation in the variation range of *d*_f_ and *d*_r_, and *J*_SC_ reaches a maximum of 12.57 mA/cm^2^ when *d*_f_ = 30 nm and *d*_r_ = 60 nm, at which case the currents in two sub-cells are matched (*J*_SC-f_ = 12.57 mA/cm^2^, *J*_SC-r_ =12.62 mA/cm^2^). Since the absorption wavelength range of PBDB-T: ITCC is relatively narrow, a thicker PBDB-T: ITCC layer is needed to match the currents generated by two sub-cells. To enhance the absorption of the PBDB-T: ITCC layer and avoid increasing its thickness, we again introduce one-dimensional rectangular wire nanogratings at the interface of PBDB-T: ITCC/Ag cathode so as to enhance the absorption in the PBDB-T: ITCC layer. Additionally, in order to reserve a certain optimization space, the thicknesses of the front and rear active layers are set as *d*_f_ = 30 nm and *d*_r_ = 45 nm. For the planar case with the same thicknesses of active layers, the currents in two sub-cells are *J*_SC-f_ = 14.78 mA/cm^2^ and *J*_SC-r_ = 9.86 mA/cm^2^ respectively, with a severe current imbalance existing in the device.

**Figure 11:**
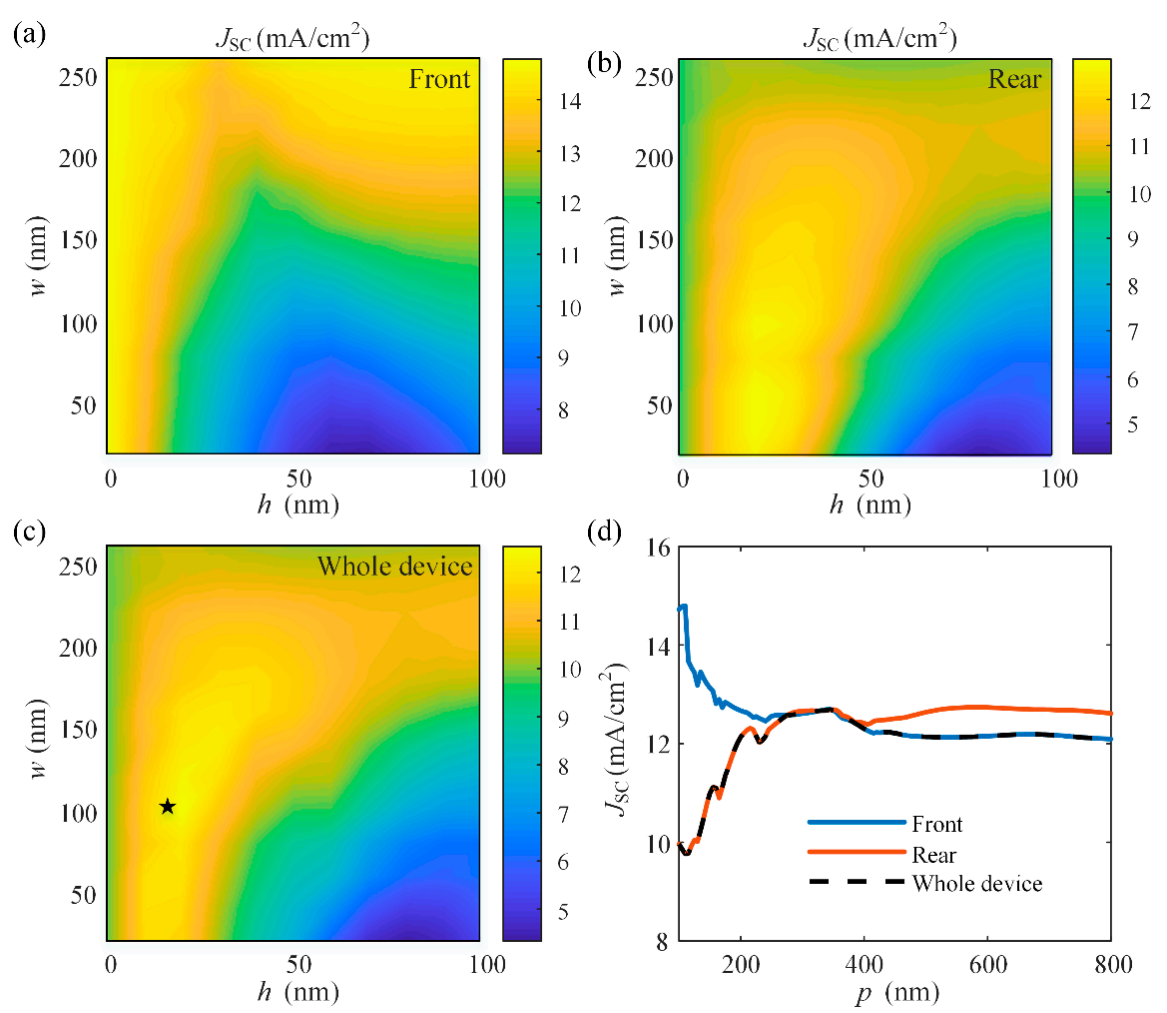
Simulated *J*_SC_ generated in (**a**) the front sub-cell, (**b**) the rear sub-cell, and (**c**) the whole tandem OSC as functions of *w* and *h* of the nanogratings at normal incidence (*p* = 300 nm) and (**d**) simulated *J*_SC_ generated in the tandem OSC as a function of *p* of the nanograting (*w* = 105 nm, *h* = 20 nm). (The star sysmbol represents the optimal structure.)

To study the effect of the nanogratings on the tandem OSC, similar to the case for the single-junction OSC, we investigated the effects of geometrical parameters of nanogratings on the device performances firstly, and the results are shown in Figure 11. The period *p* is set as 300 nm. The width *w* varies from 0 to 100 nm, and the height *h* varies from 0 to 260 nm. The *J*_SC_ generated by the rear sub-cell shows similar dependence on *w* and *h* to the case in a single device, reaching a maximum of 12.87 mA/cm^2^ when *w* = 50 nm and *h* = 20 nm. However, since more light is trapped in the rear active layer due to the effect of the nanostructures, the *J*_SC_ generated by the front sub-cell are restricted. According to Kirchhoff’s law, the *J*_SC_ of the whole tandem device is limited by the smallest *J*_SC_ of sub-cells. As a result, the *J*_SC_ of the whole device reached a maximum of 12.61 mA/cm^2^ at a width of 105 nm and a height of 20 nm. Then, as shown in Figure 11d, the period *p* is varied from 100 to 800 nm, and the *J*_SC_ maintains a relatively high value for a period longer than 210 nm and reaches a maximum of 12.69 mA/cm^2^ when *p* = 345 nm, which is improved significantly compared to the planar device (*J*_SC_ = 9.86 mA/cm^2^), even exceeding the current matched planar device (*d*_f_ = 30 nm, *d*_r_ = 60 nm) as in Figure 10b.

**Figure 12:**
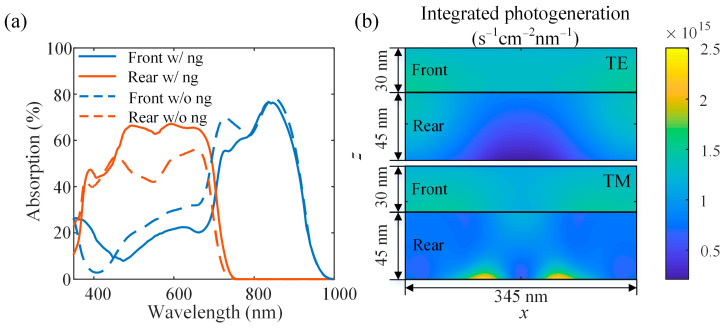
(**a**) The absorption spectra of front and rear active layers in tandem OSCs with (*w* = 105 nm, *h* = 20 nm, *p* = 345 nm) and without nanograting; (**b**) distributions of the integrated photogeneration in the front and rear active layers at different polarizations.

Figure 12 shows the comparison of the performance of the planar and non-planar tandem OSCs. The two active layer thicknesses are set as *d*_f_ = 30 nm, *d*_r_ = 45 nm, and the geometrical parameters are set as *w* = 105 nm, *h* = 20 nm, and *p* = 345 nm. The absorption spectra of the active layers in the tandem devices with and without nanogratings are shown in Figure 12a. In the planar tandem device, the front active layer has a weaker absorption than the rear active layer. There is therefore a current mismatch existed in the planar device, which limits the performance of tandem OSC a lot. Via introducing the metallic nanograting at the interface of the rear active layer and Ag cathode, the absorption of the rear active layer is enhanced significantly. Although the cathode reflected light reaching the front active layer is reduced due to the light trapping in the rear active layer, this happens to balance the current of the front and rear sub-cells and overcome the energy loss caused by the current mismatch in the planar device. Figure 12b shows the integrated photogeneration rate in two active layers at different polarizations. Similar to the case for single OSC, the obvious absorption enhancement was observed in the vicinity of the Ag grating surface at the TM polarization due to the excitation of SPP excitation, which means that more carriers are generated at the rear active layer. The above results demonstrate that the introduction of nanostructures can effectively improve the device performance of OSCs with thin active layers, both in single and tandem OSCs, and it is an effective means to balance the currents in sub-cells of tandem OSCs, avoiding increasing the thickness of the active layer with weaker absorption, which may lead to some disadvantages such as carrier combination and reduced carrier mobility.

## 4. Conclusions

In this paper, we proposed a GRCWA method to analyze and optimize the performance of OSC devices with nanostructures. In the proposed GRCWA, the effect of the incoherent glass substrate was optically modeled via a glass vector **g**. The high-order multiple reflection terms are all taken into account, which improves the accuracy of the method. For comparison, the reflectivity spectra of planar and nanostructured devices are simulated by the proposed method and existing methods. In the case of the planar OSCs, the GRCWA calculation results are well-matched with the analytical results obtained by the GTMM. For nanostructured OSCs, the proposed GRCWA can provide acceptable accuracy with less computation complexity and time compared to the ETM. With the proposed method, we simulated the absorptance, photogeneration rate distributions, and short-circuit current densities of the nanostructured devices with different geometrical parameters. The results show the potential of the GRCWA to gain insight into the optical loss and optimize the thickness and other geometrical parameters. The proposed method can provide analysis and optimization tools as well as theoretical guidance for the design procedure of OSCs. Additionally, the effect of periodic structure shape and the incident angle dependence of device performance can also be expected to be further investigated. Furthermore, we expected that the proposed optical model can provide accurate and efficient simulations for a wide range of similar hybrid coherent-incoherent optoelectric devices.

## Figures and Tables

**Table 1:** Optimized results of the single-junction OSCs with different active layer thicknesses.

Active Layer Thickness (nm)	*J*_SC_ for Planar OSCs (mA/cm^2^)	Optimized Geometrical Parameters			*J*_SC_ of Nanostructured OSCs (mA/cm^2^)
		*w* (nm)	*h* (nm)	*p* (nm)	
30	7.26	50	35	320	14.73
50	13.17	100	25	330	16.53
100	16.55	120	25	280	16.97

## Data Availability

Data sharing not applicable.

## Supplementary Materials

The following are available online https://www.mdpi.com/article/10.3390/nano11123187/s1. Figure S1: Schematic diagram of the stratified structure of the OSC with two-dimensional metal nanogratings. Figure S2: (a) Optical model of OSCs with nanostructured layer based on BSDF; (b) schematic diagram of the BSDF model for coherent multilayers with the nanostructure.
