# Supplementary material for: Optical Model and Optimization for Coherent-Incoherent Hybrid Organic Solar Cells with Nanostructures
Source: Nanomaterials (Basel). 2021 Nov 24;11(12):3187. doi: 10.3390/nano11123187 (PMC8704669; doi:10.3390/nano11123187)
Supplement: Supplementary file 1 [file nanomaterials-11-03187-s001.zip › nanomaterials-1463130-supplementary.pdf]

nanomaterials

Supplementary Materials

Optical Model and Optimization for Coherent-Incoherent Hybrid Organic Solar Cells with Nanostructures

Xuenan Zhao, Honggang Gu, Linya Chen and Shiyuan Liu

The structures of OSCs with two-dimensional nanogatings are shown in Figure S1. The periods and duty cycles of nanogratings in x and y directions are p_x, f_x and p_y, f_y, respectively. The dielectric functions of the materials in the grating area and the filling area are ε_{rd} and ε_{gr}, respectively. The dielectric function can be thought as several two-dimensional periodic functions, hence the two-dimensional Fourier expansion can be performed on the dielectric function:

$$\varepsilon(x,y)=\sum_{v=-\infty}^{\infty}\sum_{h=-\infty}^{\infty}\varepsilon_{v,h}\exp\left(\mathrm{j}\frac{2\pi v}{p_x}x\right)\exp\left(\mathrm{j}\frac{2\pi h}{p_y}y\right), \tag{S1}$$

where, the Fourier coefficient is

$$\varepsilon_{v,h}=\frac{1}{p_x p_y}\int_{-p_x/2}^{p_x/2}\int_{-p_y/2}^{p_y/2}\varepsilon(x,y)\exp\left(-\mathrm{j}\frac{2\pi v}{p_x}x\right)\exp\left(-\mathrm{j}\frac{2\pi h}{p_y}y\right)\mathrm{d}x\mathrm{d}y. \tag{S2}$$

The integers v and h indicate the expansion orders in the x and y directions, respectively.

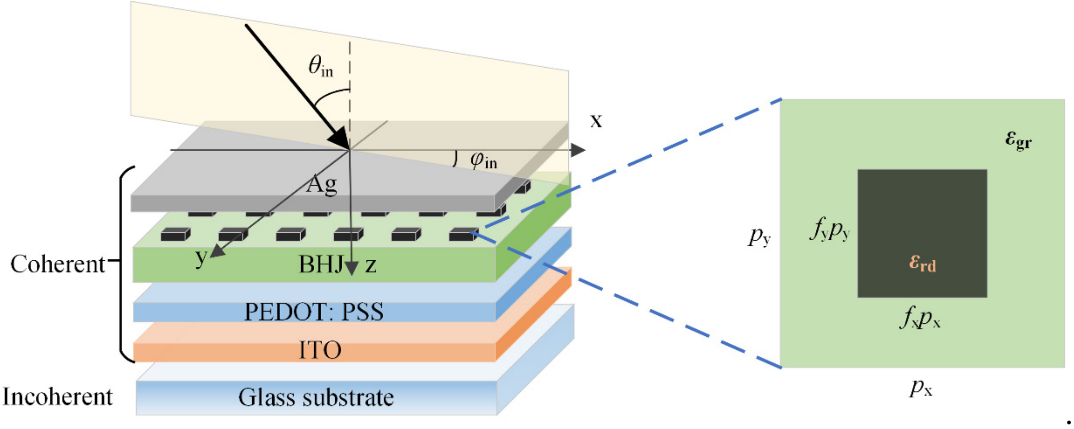

Figure S1. Schematic diagram of the stratified structure of the OSC with two-dimensional nanogratings.

For coherent region in OSC, the behavior of light propagation can be modeled based on the RCWA. According to Floquet condition, the electric and magnetic fields in grating region can be described as

$$\mathbf{E}(x,y,z)=\sum_{m=-\infty}^{\infty}\sum_{l=-\infty}^{\infty}\left[S_x^{m,l}(z)\hat{\mathrm{x}}+S_y^{m,l}(z)\hat{\mathrm{y}}+S_z^{m,l}(z)\hat{\mathrm{z}}\right]\exp\left[-\mathrm{j}\left(k_{xm}x+k_{yl}y\right)\right], \tag{S3}$$

$$\mathbf{H}(x,y,z)=\sum_{m=-\infty}^{\infty}\sum_{l=-\infty}^{\infty}\left[U_x^{m,l}(z)\hat{\mathrm{x}}+U_y^{m,l}(z)\hat{\mathrm{y}}+U_z^{m,l}(z)\hat{\mathrm{z}}\right]\exp\left[-\mathrm{j}\left(k_{xm}x+k_{yl}y\right)\right], \tag{S4}$$

where S and U are the components of the electric and magnetic fields respectively, m and l are the diffraction orders at x and y direction respectively, and $k_{xm} = k_0[n_I\sin\theta_{in}\cos\varphi_{in} - m(\lambda/p_x)]$ and $k_{yl} = k_0[n_I\sin\theta_{in}\sin\varphi_{in} - l(\lambda/p_y)]$ are the x- and y-components of the wave vector respectively.

Similar to the formulism for one-dimensional systems, substituting Equation (S3) into the Maxwell's equations and eliminating the z-components of the electric and magnetic fields, and solving the system of equations, the solution of S_x, S_y and U_x, U_y can be obtained

$$\begin{bmatrix} \mathbf{S}_y \\ \mathbf{S}_x \\ \mathbf{U}_y \\ \mathbf{U}_x \end{bmatrix} = \begin{bmatrix} \mathbf{W} & \mathbf{W} \\ -\mathbf{V} & \mathbf{V} \end{bmatrix} \begin{bmatrix} \mathbf{e}^{-k_0\mathbf{q}z} & 0 \\ 0 & \mathbf{e}^{k_0\mathbf{q}(z-d)} \end{bmatrix} \begin{bmatrix} \mathbf{C}^+ \\ \mathbf{C}^- \end{bmatrix}. \tag{S5}$$

Herein, $\mathbf{S}_x$, $\mathbf{S}_y$, $\mathbf{U}_x$, $\mathbf{U}_y$ are $ML \times 1$ vectors composed of $S_x^{m,l}$, $S_y^{m,l}$, $U_x^{m,l}$ and $U_y^{m,l}$, and M, L are the numbers of terms in the Fourier expansion of the electromagnetic field in the x and y directions, respectively.

As shown in Figure S2, the device is divided into two parts due to the thick glass substrate. The regions A and B indicate the incoherent glass substrate and coherent region, respectively. Each set of wave vectors with wavelength λ has ML orders, in other words, there are light in ML directions contemplated. Consequently, the power vectors $\mathbf{P}_1$, $\mathbf{P}_2$ would become $ML \times 1$ vectors and propagation matrices $\mathbf{R}_{gc}$, $\mathbf{T}_{gc}$, $\mathbf{R}_{ga}$, $\mathbf{T}_{ga}$ would become $ML \times ML$ matrices. The incident angle and azimuthal angle for each diffraction order are

$$\theta_r^{m,l} = \arcsin\left[\frac{1}{n_g}\left(n_a \sin\theta_{in} \cos\varphi_{in} - m\frac{2\pi}{p_x}\right)\right], \tag{S6}$$

$$\theta_t^{m,l} = \arcsin\left[\frac{1}{n_a}\left(n_a \sin\theta_{in} \cos\varphi_{in} - m\frac{2\pi}{p_x}\right)\right], \tag{S7}$$

$$\varphi_r^{m,l} = \arcsin\left[\frac{1}{n_g}\left(n_a \sin\theta_{in} \sin\varphi_{in} - l\frac{2\pi}{p_y}\right)\right], \tag{S8}$$

$$\varphi_t^{m,l} = \arcsin\left[\frac{1}{n_a}\left(n_a \sin\theta_{in} \sin\varphi_{in} - l\frac{2\pi}{p_y}\right)\right], \tag{S9}$$

The incident intensity $\mathbf{P}_2$ from region A to region B is modified as

$$\mathbf{P}_2 = T_{ag}\left[\mathbf{I} - \mathbf{R}_{gc}\mathbf{R}_{ga}\right]^{-1}\mathbf{P}_1, \tag{S10}$$

The modified reflectivity $\mathbf{R}$ of the whole device are

$$\mathbf{R} = R_{ag}\mathbf{P}_1 + \frac{T_{ag}\mathbf{T}_{ga}\mathbf{R}_{gc}}{\mathbf{I} - \mathbf{R}_{gc}\mathbf{R}_{ga}}\mathbf{P}_1 \tag{S11}$$

As for the calculation of the field and power distribution within the two-dimensional structure, it is essentially the same as for the one-dimensional structure, except that the total number of directions of light calculated changed from M to ML, and the dimensionality of the associated vectors and matrices also change.

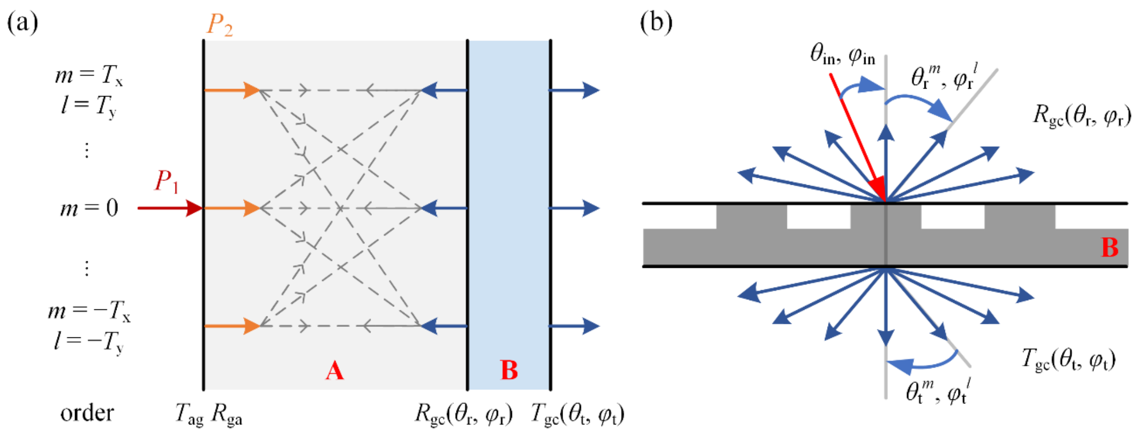

Figure S2. (**a**) Optical model of OSCs with nanostructured layer based on BSDF. Region A and B are the incoherent glass substrate and the coherent multilayers containing the nanostructured layers respectively. The grey dashed lines indicate the contribution of the reflected light at glass/air and A/B interface to each other. The reflected light of the glass/air interface at a particular order can contribute to the multi-orders reflected light at the A/B interface. While the reflected light of the A/B interface at a particular order can only contribute to the reflected light of glass/air interface at the same order. (**b**) Schematic diagram of the BSDF model for region B with nanostructured layers in Figure S2a.
